# Lipid‐Lowering Effect and Safety of Ezetimibe and Atorvastatin 5 mg in Patients With Primary Hypercholesterolemia or Mixed Dyslipidemia: A Randomized, Double‐Blind, Parallel, Multicenter, Phase 3 Clinical Trial

**DOI:** 10.1002/clc.70138

**Published:** 2025-05-13

**Authors:** You‐Jeong Ki, Weon Kim, Ki Hong Lee, Sang‐Jin Han, Yong‐Hyun Kim, Joon‐Hyung Doh, Tae Nyun Kim, Choon Hee Chung, Do Young Kim, Jin‐Man Cho, Hyuck‐Jun Yoon, In‐Kyung Jeong, Sungha Park, Kee‐Ho Song, Cheol Woong Yu, Deok‐Kyu Cho, Sung Hee Choi, Seung‐Jin Oh, Sanghoon Shin, Hyeonju Jeong, Yongwhi Park, Hyo‐Soo Kim

**Affiliations:** ^1^ Cardiovascular Center, Department of Internal Medicine Uijeongbu Eulji Medical Center Uijeongbu Republic of Korea; ^2^ Cardiovascular Division, Department of Internal Medicine Kyung Hee University Hospital, Kyung Hee University Seoul Republic of Korea; ^3^ Cardiovascular Division, Department of Internal Medicine Chonnam National University Medical School & Hospital Gwangju Republic of Korea; ^4^ Division of Cardiology, Department of Internal Medicine Hallym University Sacred Heart Hospital Anyang Republic of Korea; ^5^ Department of Endocrinology Bundang Jesaeng Hospital Bundang‐gu Gyeonggido Republic of Korea; ^6^ Inje University Ilsan Paik Hospital Goyang Republic of Korea; ^7^ Division of Endocrinology and Metabolism, Department of Internal Medicine Inje University Haeundae Paik Hospital Busan Republic of Korea; ^8^ Department of Internal Medicine and Research Institute of Metabolism and Inflammation Yonsei University Wonju College of Medicine Wonju Republic of Korea; ^9^ Division of Cardiology, Department of Internal Medicine Ajou University Hospital and Ajou School of Medicine Suwon Republic of Korea; ^10^ Department of Cardiovascular Medicine Kyung Hee University Hospital at Gangdong Seoul Republic of Korea; ^11^ Cardiovascular Center Keimyung University Dongsan Hospital Daegu Republic of Korea; ^12^ Division of Endocrinology and Metabolism, Department of Internal Medicine Kyung Hee University Hospital at Gangdong, Kyung Hee University College of Medicine Seoul Republic of Korea; ^13^ Cardiovascular Research Institute Yonsei University College of Medicine Seoul Republic of Korea; ^14^ Division of Endocrinology and Metabolism Konkuk University Medical Center, Konkuk University School of Medicine Republic of Korea; ^15^ Department of Cardiology, Cardiovascular Center Korea University Anam Hospital Seoul Republic of Korea; ^16^ Yongin Severance Hospital Yonsei University College of Medicine Republic of Korea; ^17^ Department of Internal Medicine, Seoul National University College of Medicine Seoul National University Bundang Hospital Seongnam‐City Republic of Korea; ^18^ Division of Cardiology National Health Insurance Service Ilsan Hospital Goyang Republic of Korea; ^19^ Division of Cardiology, Department of Internal Medicine Ewha Womans University Seoul Hospital Seoul Republic of Korea; ^20^ Division of Cardiology, Department of internal medicine, Myongji Hospital Hanyang University Medical Center Goyang Republic of Korea; ^21^ Division of Cardiology, Department of Internal Medicine Gyeongsang National University Changwon Hospital Changwon Republic of Korea; ^22^ Cardiovascular Center, Department of Internal Medicine Seoul National University Hospital Seoul Republic of Korea

**Keywords:** atorvastatin, cholesterol, ezetimibe, statin

## Abstract

**Objective:**

This study aimed to compare the lipid‐lowering effect and safety of low‐intensity atorvastatin (5 mg) plus ezetimibe (10 mg) combination therapy (A5E10) with monotherapy regimens–atorvastatin 5 mg [A5], ezetimibe 10 mg [E10], and atorvastatin 10 mg [A10])–in dyslipidemia patients.

**Methods:**

A randomized, double‐blind, placebo‐controlled trial involving 252 dyslipidemia patients was conducted at 25 centers in South Korea (NCT05970679). Participants aged ≥ 19 years were randomized into four groups: A5E10, A5, E10, and A10. The primary endpoint was the percentage change in low‐density lipoprotein cholesterol (LDL‐C) levels from baseline to 8 weeks. Secondary endpoints included changes in other lipid parameters, lipid ratios, LDL‐C goal achievement rates and safety assessments.

**Results:**

The mean age of the patients was 63 years, and 51.2% were male. The A5E10 group showed significantly greater LDL‐C reduction (47.6%) compared with A5 (33.4%), E10 (19.4%), and A10 (40.1%) at 8 weeks (*p* < 0.0001). A5E10 also significantly reduced triglyceride, non‐high‐density lipoprotein cholesterol, and apolipoprotein B levels. In addition, a significant reduction in LDL‐C levels was observed over the 4 weeks, with a 46.7% reduction in LDL‐C levels after 4 weeks of A5E10 administration. No severe adverse events were observed in the A5E10 group.

**Conclusion:**

The combination of low‐intensity atorvastatin and ezetimibe was more effective than moderate‐intensity atorvastatin monotherapy in lowering LDL‐C levels and improving other lipid parameters. It was well‐tolerated and demonstrated rapid benefits within a month, offering a promising alternative for patients with low to moderate cardiovascular risk who do not achieve adequate control with statin monotherapy.

## Graphical abstract

- The combination of low‐dose atorvastatin (5 mg) and ezetimibe (10 mg) significantly reduced LDL‐C levels by 47.6%, outperforming monotherapies in patients with dyslipidemia.
- This combination therapy showed rapid efficacy, achieving a 46.7% reduction in LDL‐C within just 4 weeks of treatment.
- In addition to lowering LDL‐C, the therapy improved other lipid parameters, including non‐HDL‐C and Apo B, offering a comprehensive lipid‐lowering effect with a favorable safety profile.

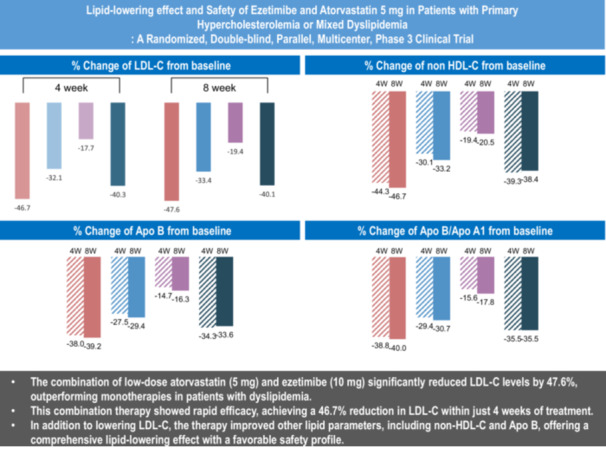

**Abbreviations**
ADRs: adverse drug reactions
Apo AI: apolipoprotein AI
Apo B: apolipoprotein B
CK: creatine kinase
CVD: cardiovascular disease
HDL‐C: high‐density lipoprotein cholesterol
hs‐CRP: high‐sensitivity C‐reactive protein
IDL: intermediate‐density lipoprotein
LDL‐C: low‐density lipoprotein cholesterol
SAEs: serious adverse events
SDs: standard deviations
TC: total cholesterol
TG: triglyceride
ULN: upper limit normal
VLDL: very low‐density lipoprotein

## 1 Introduction

Dyslipidemia is an important and modifiable risk factor for cardiovascular disease (CVD) [1], which remains the leading cause of mortality worldwide. In particular, elevated low‐density lipoprotein cholesterol (LDL‐C) levels, are strongly associated with an increased risk of atherosclerosis and subsequent cardiovascular events [2]. According to the meta‐analysis, the lowering of LDL‐C by about 38.7 mg/dL (1 mmol/L) with statin regimens safely reduced the major coronary events about a 23% [3]. Consequently, lipid‐lowering therapies are key components in the prevention and treatment of CVD [1, 2].

Hydroxymethylglutaryl‐coenzyme A reductase inhibitors, commonly known as statins, are widely prescribed for their effectiveness in reducing LDL‐C levels and improving cardiovascular outcomes [1, 4, 5, 6]. Importantly, aggressive LDL‐C lowering has been shown to stabilize atherosclerotic plaques, reduce inflammation, and ultimately prevent clinical events [7, 8]. However, despite the proven efficacy of statins, some patients do not achieve their target LDL‐C levels or experience side effects associated with higher doses [9, 10]. The most common adverse effects of high‐intensity statins include myopathy, an increased risk of hepatotoxicity, and new‐onset diabetes [11, 12, 13].

Atorvastatin, a widely used statin, has demonstrated significant LDL‐C lowering effects and a reduction in cardiovascular events across various risk populations [14]. Consequently, combination therapies that integrate supplementary lipid‐lowering agents, such as ezetimibe, are gaining interest. Ezetimibe, a cholesterol absorption inhibitor, has shown promise in further reducing LDL‐C levels when added to statin therapy, providing a potential strategy for patients who do not reach their lipid targets with statin monotherapy [15, 16]. These findings support the complementary mechanisms of action and the potential of combining atorvastatin and ezetimibe to achieve effective LDL‐C lowering and improve clinical outcomes, particularly in patients with primary hypercholesterolemia or mixed dyslipidemia. Although guidelines recommend a moderate‐intensity statin that can lower LDL‐C level by 30%–49% for effective primary prevention in patients at low to moderate cardiovascular risk, there are limited studies comparing the effectiveness and safety of low‐intensity atorvastatin and ezetimibe combination therapy to moderate‐intensity statin [17, 18]. In the RACING trial, the combination of a moderate‐intensity statin and ezetimibe was non‐inferior to high‐intensity statin therapy on the risk of CVD in patients with atherosclerotic cardiovascular disease [19]. However, it is not known whether a low‐intensity statin plus ezetimibe is an alternative to a moderate‐intensity statin in the general population. Thus, in this study, we aimed to compare the lipid‐lowering effect and safety of a combination therapy of atorvastatin 5 mg and ezetimibe 10 mg (A5E10) with monotherapy regimens (atorvastatin 5 mg [A5], ezetimibe 10 mg [E10], or atorvastatin 10 mg [A10]) in patients with dyslipidemia.

## 2 Methods

### 2.1 Study Design

This randomized, double‐blind, placebo‐controlled trial was conducted at 25 centers in South Korea to compare the effects of A5E10 combination therapy with A5, E10, and A10 monotherapies on lipid levels, lipid parameter ratios, LDL‐C target achievement rates, and safety profiles in patients with dyslipidemia (NCT05970679). This study was conducted from July 2022 to April 2023. After the initial screening, patients who met the inclusion and exclusion criteria at visit 1 received a placebo to assess drug compliance and were instructed to discontinue any lipid‐lowering medications for at least 4 weeks (6 weeks for fibrate) (Figure S1, and Table S1). Throughout the study period, all patients received therapeutic lifestyle interventions. Following a run‐in period, the patients were re‐screened at visit 2 based on their lipid profile and inclusion/exclusion criteria. Eligible patients were stratified according to their cardiovascular risk category at visit 2 and randomized into one of the four treatment groups (Table S2). Risk factors and lipid levels were used to determine cardiovascular disease risk categories according to the 2018 Guidelines for the Management of Dyslipidemia in Korea [20]. Efficacy and safety assessments were conducted 4 and 8 weeks after randomization.

### 2.2 Participants

Participants were eligible for inclusion in this study if they had dyslipidemia at age ≥ 19 years, provided written informed consent, and had fasting LDL‐C level ≤ 250 mg/dL and triglyceride (TG) level < 500 mg/dL. They also had to have LDL‐C and TG levels in the risk category at visit 2 and demonstrate medication adherence between 70% and 130% during the run‐in period. The following patients were excluded from this study: patients with severe heart failure (New York Heart Association class III or IV); with a history of acute coronary syndrome, ischemic stroke, or coronary revascularization within the last 3 months; with uncontrolled hypertension (systolic blood pressure ≥ 180 mmHg or diastolic blood pressure ≥ 110 mmHg); with type 1 diabetes or uncontrolled type 2 diabetes (hemoglobin A1c ≥ 9%); with thyroid dysfunction (thyroid‐stimulating hormone ≥ 1.5 × upper limit normal [ULN]); with malignant tumors within the last 5 years; with impaired drug absorption, distribution, metabolism, or excretion; secondary dyslipidemia; with a history of alcohol abuse; with a history of myopathy, rhabdomyolysis, or fibromyalgia; with hypersensitivity to atorvastatin or ezetimibe; who were pregnant or breastfeeding; or with laboratory abnormalities, including creatine kinase (CK) ≥ 2 × ULN, estimated glomerular filtration rate by Modification of Diet in Renal Disease < 30 mL/min/1.73 m^2^, or aspartate transaminase or alanine aminotransferase ≥ 2 × ULN.

### 2.3 Randomization

Stratified block randomization was performed to allocate the participants to treatment groups based on their cardiovascular risk categories. Daewon Pharmaceutical Co. Ltd. prepared and provided investigational drugs according to a randomization list. Each participant was prescribed four pills daily for 8 weeks, one containing the actual medication and three placebos. Both patients and researchers were blinded to the group allocation, and all patients adhered to therapeutic lifestyle changes during the study period.

### 2.4 Study Endpoints

The primary efficacy endpoint was the percentage change in LDL‐C levels from baseline to the 8‐week follow‐up period. The secondary efficacy endpoints included the following variables: (1) percentage change in LDL‐C levels from baseline to the 4‐week follow‐up; (2) percentage change in lipid profile components, including total cholesterol (TC), TG, high‐density lipoprotein cholesterol (HDL‐C), non‐HDL‐C, apolipoprotein AI (Apo AI), and apolipoprotein B (Apo B) levels from baseline to the 4‐ and 8‐week follow‐ups; (3) percentage change of lipid parameter ratios, including LDL‐C/HDL‐C, TC/HDL‐C, non‐HDL‐C/HDL‐C, and Apo B/Apo AI, from baseline to 4‐ and 8‐week follow‐ups; (4) LDL‐C goal achievement rates at 4‐ and 8‐weeks according to cardiovascular risk category; and (5) percentage change in high‐sensitivity C‐reactive protein (hs‐CRP) and fibrinogen levels from baseline to 4‐ and 8‐week follow‐ups. The safety analysis was based on the safety set. Adverse events, clinical evaluations, laboratory data, and electrocardiograms were monitored as safety endpoints. The number of cases and incidence rates for all treatment‐emergent adverse events, adverse drug reactions (ADRs), serious adverse events (SAEs), and serious ADRs are presented according to the system organ class and preferred terms.

### 2.5 Statistical Analyses

Efficacy endpoints were analyzed using intention‐to‐treat analysis, and safety endpoints were analyzed using per‐protocol analysis. The full analysis set was used for baseline characteristics and efficacy assessment, whereas the safety analysis set was used for safety assessment. The full analysis included participants who underwent at least one primary endpoint assessment, and the safety set included all participants who were administered the investigational medication. Gatekeeping procedures were implemented to evaluate the efficacy endpoints to reduce the risk of type I errors. This involved a two‐stage process. Stage 1 tested the superiority of treatment group A5 over control group E10. If superiority was confirmed, we proceeded to the second stage in which we tested the superiority of A5E10 over both E10 and A5. In other words, the null hypothesis had to be rejected in the first test before the second test could be conducted. The sample size calculation determined that 240 patients (60 patients per group) were required to achieve an overall power of ≥ 95% combined with a 15% attrition rate, assuming a weighted mean difference in LDL‐C of 13.9% with a standard deviation of 18.6% from a conservative perspective at stage 2 for the comparison between A5E10 and A5 [18, 21].

Numerical data are expressed as means ± standard deviations (SDs) for continuous variables and as percentages for categorical variables. Continuous variables were compared using Student's t‐test and the Kruskal–Wallis H test when they were normally and non‐normally distributed, respectively. Categorical variables were compared using the χ2 test or Fisher's exact test. Differences within groups were compared using the paired t‐test or Wilcoxon signed‐rank test. The difference in the percentage change (%) from baseline to 8 weeks between the groups was assessed using an analysis of covariance (ANCOVA), adjusting for baseline laboratory values and cardiovascular risk categories as covariates. A two‐sided *p* < 0.05 was considered significant. All the statistical analyses were performed using SAS version 9.4 (SAS Institute Inc., Cary, NC, USA).

## 3 Results

### 3.1 Baseline Characteristics

**Figure 1:**
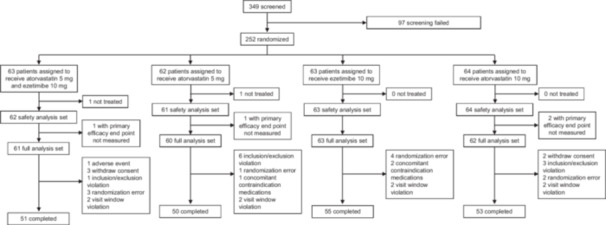
Study flow.

A total of 349 patients were screened, of whom 252 were randomized into one of four treatment groups: A5E10 (*n* = 63), A5 (*n* = 62), E10 (*n* = 63), and A10 (*n* = 64). The first patient was enrolled in July 2022, and the last patient was observed in April 2023. Of the randomized patients, two were excluded from the safety set because they did not take the investigational medication, leaving 250 patients for the safety analysis. For the efficacy evaluation, four patients did not undergo efficacy evaluation, resulting in 246 patients being included in the full analysis set, and 209 patients were included in the per‐protocol set (Figure 1). The baseline characteristics of the enrolled patients are shown in Table 1. The mean (±SD) age of the participants was 63.0 ± 10.8 years, and 51.2% of the participants were male. At the time of randomization, 164 (66.7%) patients had hypertension, and 109 (44.3%) had diabetes. There were no statistically significant differences in the baseline demographic data between the treatment groups, except for smoking status (*p* = 0.049).

**Table 1:** Baseline characteristics.

Characteristics	Atorvastatin 5 mg/Ezetimibe 10 mg (*n* = 61)	Atorvastatin 5 mg (*n* = 60)	Ezetimibe 10 mg (*n* = 63)	Atorvastatin 10 mg (*n* = 62)
Age, years	62.0 ± 10.5	64.6 ± 10.3	62.4 ± 12.0	63.0 ± 10.3
Male, *n* (%)	34 (55.7)	26 (43.3)	34 (54.0)	32 (51.6)
Height, cm	164.1 ± 8.6	160.9 ± 7.9	162.0 ± 7.9	162.5 ± 7.7
Body weight, kg	67.4 ± 13.9	64.1 ± 11.4	66.9 ± 10.7	67.5 ± 8.7
Risk factors, *n* (%)				
Hypertension	40 (65.6)	43 (71.7)	38 (60.3)	43 (69.4)
Diabetes	34 (55.7)	26 (43.3)	23 (36.5)	26 (41.9)
Current smoker	13 (21.3)	4 (6.7)	10 (15.9)	9 (14.5)
Medical history, *n* (%)				
Asymptomatic CAD	8 (13.1)	9 (15.0)	9 (14.3)	8 (12.9)
Angina	16 (26.2)	19 (61.7)	20 (3.2)	18 (29.0)
MI	5 (8.2)	6 (10.0)	6 (9.5)	5 (8.1)
Cerebral infarction	3 (4.9)	2 (3.3)	1 (1.6)	3 (4.8)
Carotid artery stenosis	0 (0)	0 (0)	2 (3.2)	2 (3.2)
Peripheral artery disease	3 (4.9)	0 (0)	2 (3.2)	3 (4.8)
Heart failure	1 (1.6)	4 (6.7)	2 (3.2)	6 (9.7)
Risk category, *n* (%)*				
Low	5 (8.2)	7 (11.6)	7 (11.1)	7 (11.3)
Moderate	9 (14.8)	9 (15.0)	9 (14.3)	9 (14.5)
High	18 (29.5)	16 (26.7)	18 (28.6)	18 (29.0)
Very high	29 (47.5)	28 (46.7)	29 (46.0)	28 (45.2)

*Note:* Data presented as mean ± standard deviation or as n (%).

Abbreviations: CAD, coronary artery disease; MI, myocardial infarction.

* Risk factors and lipid levels were used to determine cardiovascular disease risk categories according to the 2018 Guidelines for the Management of Dyslipidemia in Korea.

### 3.2 Efficacy Outcomes

**Figure 2:**
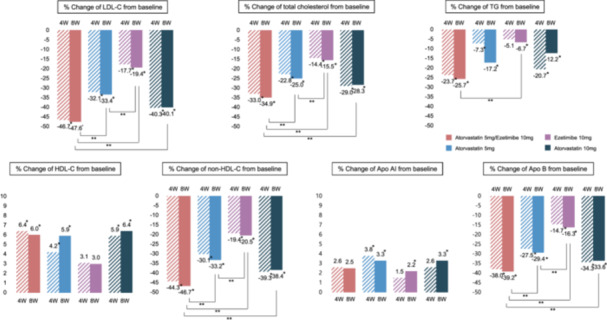
Percentage changes in lipid profile at 4 and 8 weeks follow‐up. *In group *p*‐value < 0.0001, **Between group *p*‐value < 0.0001. Apo AI, apolipoprotein A‐I; Apo B, apolipoprotein B; HDL‐C, high density lipoprotein cholesterol; LDL‐C, low density lipoprotein cholesterol; non‐HDL‐C, non–high‐density lipoprotein cholesterol; TG, triglyceride.

**Figure 3:**
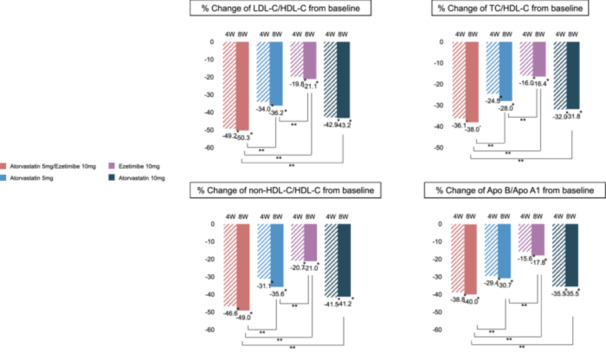
Percentage changes in lipid parameters ratios at 4 and 8 weeks follow‐up. *In group *p*‐value < 0.0001, **Between group *p*‐value < 0.0001. Abbreviations as in Figure 3.

Figure 2 and Table S3 summarize the changes in lipid level, lipoprotein level, lipid parameter ratios, and hs‐CRP, and fibrinogen levels. The A5E10 group demonstrated a significantly greater reduction in LDL‐C levels (47.6%) than the A5 (33.4%, between groups *p* < 0.0001), E10 (19.4%, between groups *p* < 0.0001), and A10 (40.1%, between groups *p* < 0.0001) groups after 8 weeks of treatment. Additionally, a significant reduction (46.7%) in LDL‐C levels was observed at 4 weeks after A5E10 administration. The A5E10 group also had lower TG levels (25.7%) compared with the E10 group (6.7%, between groups *p* < 0.0001). However, changes in HDL‐C and Apo AI levels were not significantly different between the groups. All lipid parameter ratios, including LDL‐C/HDL‐C, TC/HDL‐C, non‐HDL‐C/HDL‐C, and Apo B/Apo AI, also changed significantly after 4 weeks of treatment, with a significant decrease in the A5E10 group compared with the other groups at 8 weeks (Table S3 and Figure 3).

Figure S2 shows the target LDL‐C achievement rates according to cardiovascular risk categories. The A5E10 group had a significantly higher rate of achieving the target LDL‐C levels than the other three groups. Overall, the target LDL‐C achievement rate in the A5E10 group was 67.2% at 4 weeks and remained stable at 8 weeks. Specifically, the LDL‐C target was achieved in 44.8%, 77.8%, and 100% of the patients in the very high‐risk, high‐risk, and low‐ to moderate‐risk groups, respectively.

### 3.3 Safety Outcomes

A total of 51 adverse events occurred in 43 of the 250 patients (17.2%) in the safety set, with no significant differences observed among the four treatment groups (*p* = 0.913, Table 2 and Table S4). Among these, two were classified as severe: an ulna fracture in the A5 group and spinal pain in the E10 group; both were determined to be unrelated to the medication. In addition, eight ADRs occurred in 7 (2.8%) patients, with no statistically significant differences between the groups. Two adverse events led to discontinuation of the medication: elevated blood CK level in the A5E10 group and dizziness in the A10 group. Importantly, no patient experienced significant increases in liver transaminase (> 3 times the ULN) or CK levels (> 10 times the ULN) during the study.

**Table 2:** Treatment‐related side effects.

Characteristics	Atorvastatin 5 mg/Ezetimibe 10 mg (*n* = 62)	Atorvastatin 5 mg (*n* = 61)	Ezetimibe 10 mg (*n* = 63)	Atorvastatin 10 mg (*n* = 64)
Treatment emergent adverse event (patients)	11 (17.7)	11 (18.0)	9 (14.3)	12 (18.8)
Adverse drug reaction (patients)	2 (3.2)	1 (1.6)	1 (1.6)	3 (4.7)
Reported adverse drug reaction				
Gastrointestinal disorders	1 (1.6)	0 (0)	1 (1.6)	1 (1.6)
Myalgia	0 (0)	1 (1.6)	0 (0)	1 (1.6)
Dizziness	0 (0)	0 (0)	0 (0)	2 (3.1)
Creatine phosphokinase increased	1 (1.6)	0 (0)	0 (0)	0 (0)
Serious adverse event (patients)	0 (0.0)	1 (1.6)	1 (1.6)	0 (0)
Serious adverse drug reaction (patients)	0 (0)	0 (0)	0 (0)	0 (0)
Adverse event leading to withdrawal (patients)	1 (1.6)	0 (0)	0 (0)	1 (1.6)

*Note:* Values are presented as the number of patients experiencing each event (n) and the corresponding percentage of the total group (%). Multiple events occurring in the same patient were counted separately.

## 4 Discussion

This study assessed the lipid‐lowering effect and safety of combination therapy with atorvastatin (5 mg) and ezetimibe (10 mg) compared with monotherapy regimens in patients with dyslipidemia. The key findings of this study are as follows. (1) The A5E10 combination therapy reduced LDL‐C level by 47.6% from baseline LDL‐C after 8 weeks of treatment, which was significantly greater than the reductions achieved with atorvastatin 5 mg, ezetimibe 10 mg, or atorvastatin 10 mg monotherapy. (2) A5E10 combination therapy demonstrated a rapid onset of action, with a 46.7% reduction in LDL‐C levels observed as early as 4 weeks. By week 4, combination therapy also led to a 23.7% reduction in TG levels, a 44.3% reduction in non‐HDL‐C levels, and a 38.0% reduction in Apo B levels. (3) All lipid parameter ratios, including LDL‐C/HDL‐C, TC/HDL‐C, non‐HDL‐C/HDL‐C, and Apo B/Apo AI, showed significant changes after 4 weeks of treatment, with the most pronounced reductions observed in the A5E10 group at 8 weeks. Notably, no SAEs were observed in the combination therapy group.

### 4.1 The Benefits of Low‐Intensity Statin Combination Therapy Over Moderate‐Intensity Statin Therapy

Lowering LDL‐C improves clinical outcomes and statins are recommended as the mainstay of lipid‐lowering therapy. Therefore, recent lipid guidelines recommend LDL‐C reduction based on cardiovascular risk. Clinical guidelines recommend moderate‐intensity statins for primary prevention in adults aged 40–75 years without diabetes and at a moderate cardiovascular risk, or in patients with diabetes and aged 40–75 years [22]. Although the initial lowering of LDL‐C levels with statins is effective, further reductions require disproportionately larger dose increases. This is because statin‐induced reductions in serum cholesterol can lead to increased cholesterol absorption from the gastrointestinal tract, which diminishes the efficacy of higher statin doses [23]. Typically, doubling the statin dose from the minimum effective dose yields only an additional 6% reduction in serum LDL‐C concentration [23]. In addition, side effects associated with statins include myopathy, new‐onset diabetes, and an increased risk of hepatotoxicity [11, 12, 13]. The SEARCH trial compared the effects of 80 mg versus 20 mg of simvastatin in patients with a history of myocardial infarction [24]. The trial demonstrated a higher risk of myopathy in high‐dose simvastatin (0.9%) compared to the low‐dose group (0.03%). Similarly, the JUPITER trial, which compared rosuvastatin 20 mg to placebo, reported a higher incidence of new‐onset diabetes in the rosuvastatin (3.0% vs. 2.4%) [25]. In another meta‐analysis comparing high‐intensity and moderate‐intensity statins, new‐onset DM occurred 12% more often in the high‐intensity statin group compared with the moderate‐intensity statin group [26]. These findings highlight the dose‐dependent manner in which statin‐associated side effects, which are important because they reduce patient adherence, are associated with clinical outcomes. Our study found that combining a low‐intensity statin atorvastatin 5 mg with ezetimibe resulted in a 47.6% reduction in LDL‐C from baseline, a significantly greater reduction than that achieved with atorvastatin 10 mg monotherapy, which is classified as a moderate‐intensity statin. Moreover, in the A5E10 group, 100% of participants with low‐to‐moderate risk achieved their LDL‐C targets. Consistent with the results of previous studies [17, 18], these findings suggest that adding ezetimibe to a low‐intensity statin can achieve greater LDL‐C reduction than a moderate‐intensity statin alone, potentially offering a more effective and better‐tolerated treatment option by reducing the side effects commonly associated with high‐intensity statin therapy [10].

Side effects associated with statins can be increased by drug interactions and major comorbidities that are more common in older people. Statin combination preparations may be more beneficial than high‐dose statins in elderly patients. In the age‐stratified post‐hoc analysis of the RACING trial, ezetimibe combination therapy was associated with a lower rate of intolerance related drug discontinuation and new‐onset diabetes than high‐intensity statin monotherapy in patients aged ≥ 75 years [27]. In this respect, low intensity statin with ezetimibe combination therapy may be a good alternative for the treatment of hyperlipidemia in older people who are not at high risk. Moreover, previous studies have shown that Asian populations tend to achieve comparable LDL‐C reductions with lower statin doses than Western populations [28, 29]. For instance, the Japanese Lipid Intervention Trial (J‐LIT) and the MEGA study demonstrated that low‐dose statin therapy significantly reduced both LDL‐C levels and coronary heart disease risk in Japanese patients, with efficacy similar to that of higher statin doses used in Western cohorts [28, 29]. These findings provide further support for the clinical utility of low‐intensity statin plus ezetimibe therapy in Asian patients with dyslipidemia.

### 4.2 Time to Effect of the Combination of a Statin Plus Ezetimibe

Most previous randomized trials of statin treatment have assessed on evaluating the extent of LDL‐C reduction after 1 year of treatment [5, 28, 30, 31]. However, the PROVE IT‐TIMI 22 trial demonstrated significant LDL‐C reductions within just 1 month, with reductions of 51% in the atorvastatin 80 mg group and 22% in the pravastatin 40 mg group. This highlights the rapid onset of action of atorvastatin, with another study showing that approximately 90% of the maximum LDL‐C reduction from baseline was achieved within the first 2 weeks of treatment [32]. In our study, the combination therapy (A5E10) produced remarkable lipid‐lowering effects, including 98% of the maximum LDL‐C reduction, 92% of the maximum TG reduction, 95% of the maximum non‐HDL‐C reduction, and 97% of the maximum Apo B reduction in just 1 month. Such a rapid action is critical for patients requiring swift LDL‐C reduction to mitigate cardiovascular risks.

### 4.3 Effect of Combination of a Statin Plus Ezetimibe on Apolipoprotein Ratios Other Than Low‐Density Lipoprotein Cholesterol Levels

The primary goal in treating dyslipidemia is to reduce LDL‐C levels according to cardiovascular risk [2], and other components of the lipoprotein‐lipid profile also contribute to the residual CVD risk [5]. Other lipoprotein subsets that transport cholesteryl esters in the circulation, such as very low‐density lipoprotein (VLDL), intermediate‐density lipoprotein (IDL), and chylomicron remnants, are also proatherogenic. Therefore, all cholesteryl esters carried by lipoproteins other than HDL (non‐HDL‐C), TG levels, or the ratio of total cholesterol to HDL‐C also predict cardiovascular events [33]. Apolipoprotein, embedded in the phospholipid bilayer of the lipoproteins, play a crucial role in determining their function. HDL‐C particles primarily contain Apo AI and Apo AII, whereas LDL‐C particles are mainly associated with Apo B [34]. Although HDL‐C has been traditionally associated with a protective effect against cardiovascular risk [35], it is important to note that HDL functionality, rather than concentration, is more strongly predictive of cardiovascular outcomes [36, 37]. HDL particles exert various atheroprotective functions, including reverse cholesterol transport, antioxidant, and anti‐inflammatory effects, which are not fully captured by HDL‐C levels alone [36]. Measuring Apo B provides more comprehensive clinical information than measuring LDL‐C alone, as Apo B reflects the presence of other atherogenic lipoproteins, such as VLDL and IDL, in addition to LDL‐C. The AMORIS study showed that Apo B, Apo AI, and the ratio of Apo B/Apo AI were important factors in predicting fatal MI, and Apo B was more important than LDL‐C in predicting fatal myocardial infarction [38]. Additionally, the Apo B/Apo AI ratio, representing the balance between atherogenic and atheroprotective lipoproteins, is more strongly correlated with the risk of ischemic heart disease than traditional lipid parameters [3, 38, 39]. In our study, after 2 months of treatment with the A5E10 combination therapy, we observed a 39.2% reduction in Apo B levels and a 40% reduction in the Apo B/Apo AI ratio. These findings underscore the potential of combination therapy to not only reduce LDL‐C but also favorably modulate other atherogenic lipoprotein ratios, thereby potentially lowering the residual risk of cardiovascular events beyond that achieved by LDL‐C reduction alone.

## 5 Limitations

Our study has some limitations. First, although this study was adequately powered for the primary endpoints, the sample size was relatively small, and this study was conducted across a limited number of centers in South Korea. This may limit the generalizability of the findings to wider populations of different ethnicities or healthcare settings. Second, the short‐term duration of this study limits the evaluation of long‐term efficacy, safety, and cardiovascular outcomes. In addition, this therapy may not be suitable for patients with acute coronary syndrome who require immediate and intensive LDL‐C lowering. Third, the exclusion criteria, particularly the exclusion of patients with severe CVD or uncontrolled comorbidities, may have resulted in a study population that was not fully representative of the real‐world dyslipidemia population, potentially limiting the applicability of the findings to high‐risk patients. Fourth, the study included patients with a wide range of baseline risk, including 76% of patients who were at high or very high risk. Fixed‐dose combination of low‐intensity atorvastatin and ezetimibe has moderate efficacy in reducing LDL‐C by 30% to 50%, and our results may not apply to patients at high or very high risk. Fifth, HDL‐C functionality, which may offer additional insights beyond HDL‐C concentration in predicting cardiovascular risk, was not assessed in this study [36, 37]. This aspect could be considered in future investigations to further understand the potential impact of combination therapy on HDL‐related effects. Finally, the risk categories and LDL‐C targets of the participants in this study were based on the Korean Guidelines for the Management of Dyslipidemia, 4th edition [20]. Differences in future guidelines may affect the interpretation and application of the results.

## 6 Conclusion

The combination of low‐intensity atorvastatin (5 mg) and ezetimibe (10 mg) is significantly more effective in reducing LDL‐C levels than low‐intensity atorvastatin (5 mg), ezetimibe (10 mg), or moderate‐intensity atorvastatin (10 mg) monotherapy in patients with dyslipidemia. The combination therapy also shows good efficacy in improving other lipid levels, including non‐HDL‐C and Apo B levels, and the Apo B/Apo AI ratio, with a rapid onset of the effect observed within the first 4 weeks of treatment. This approach offers a viable alternative for patients who are intolerant to high‐intensity statins or who are unable to use or afford PCSK9 inhibitors.

## Author Contributions

Hyo‐Soo Kim contributed to the design and implementation of the study, the analysis of results, manuscript writing, critical revision, funding acquisition, and supervision of the research. You‐Jeong Ki and Weon Kim were responsible for data interpretation and drafting the manuscript. All authors, except You‐Jeong Ki, participated in data collection, supervised the trial, and revised the manuscript for important intellectual content.

## Conflicts of Interest

The authors declare no conflicts of interest.

## Supporting information

AtoEze supplementary ver4.

## Data Availability

The data that support the findings of this study are available on request from the corresponding author. The data are not publicly available due to privacy or ethical restrictions.
