# Supplementary material for: Lipid‐Lowering Effect and Safety of Ezetimibe and Atorvastatin 5 mg in Patients With Primary Hypercholesterolemia or Mixed Dyslipidemia: A Randomized, Double‐Blind, Parallel, Multicenter, Phase 3 Clinical Trial
Source: Clin Cardiol. 2025 May 13;48(5):e70138. doi: 10.1002/clc.70138 (PMC12070249; doi:10.1002/clc.70138)
Supplement: Supplementary file 1 — AtoEze supplementary ver4. [file CLC-48-e70138-s001.docx]

**SUPPLEMENTARY MATERIALS**

**Supplementary Table 1. Inclusion and exclusion criteria**

**Inclusion criteria**

1. Patients aged ≥19 years
2. Patients diagnosed with primary hypercholesterolemia or mixed dyslipidemia
3. Patients with LDL-C ≤250mg/dL, and Triglycerides <500 mg/dL at Visit 1
4. Patients who met the specified criteria for each group were identified based on their fasting serum lipid measurements and cardiovascular risk factors at the time of randomization

| **Risk category^*^** | **LDL-C (mg/dL)** | **TG (mg/dL)** |
| --- | --- | --- |
| Low-risk group (at least one of the risk factor^**^) | 160-250 | <500 |
| Moderate-risk group (two or more risk factors) | 130-250 |  |
| High-risk group  - Carotid artery disease  - Abdominal aortic aneurysm  - Diabetes | 100-250 |  |
| Very high-risk group  - Coronary artery disease  - Atherosclerotic ischemic stroke and transient cerebral ischemic attack  - Peripheral artery disease | 70-250 |  |

^*^ Based on the Korean Guidelines for the Management of Dyslipidemia 4th edition

^**^The major risk of cardiovascular diseases except LDL-cholesterol

- Age: male ≥45 years, female ≥55 years (at Visit 1)
- Family history of premature atherosclerotic cardiovascular disease (male <55 years, female <65 years) (at Visit 1)
- Hypertension: SBP ≥140 mmHg or DBP ≥90 mmHg or taking antihypertensive drugs (at Visit 3)
- Smoking (at Visit 1)
- Low HDL-C: HDL-C <40 mg/dL (at Visit 2)
- High HDL-C (≥60mg/dL) is considered as a protective factor, and one factor is excluded from the number of risk factors (at Visit 2)

1. Patients with medication adherence confirmed ≥70% and ≤130% during the run in period
2. Patients who voluntarily provided written consent to participate in this clinical trial

**Exclusion criteria**

1. Patients with any of the following laboratory abnormalities or conditions
   1. Patients with a medical or family history of myopathy, rhabdomyolysis, fibromyalgia, hereditary myopathy, or CK (CPK) levels ≥2 X upper limit of normal (ULN) at Visit 1 laboratory results
   2. Patients with eGFR (MDRD) <30 mL/min/1.73 m^2^ at Visit 1 laboratory results
   3. Patients with active liver disease, active or chronic hepatobiliary disease, or serum ALT or AST levels ≥2 X ULN
   4. Patients with uncontrolled hypertension (SBP ≥180mmHg, or DBP ≥110mmHg), Type 1 DM, uncontrolled Type 2 DM (HbA1c ≥9%), or uncontrolled thyroid function (TSH ≥1.5 X ULN)
2. Patients with a confirmed medical or treatment history of any of the following
   1. Patients with severe heart failure (NYHA class III, or IV)
   2. Patients diagnosed with acute coronary syndrome or cerebrovascular disease or with a history of percutaneous coronary intervention or coronary artery bypass graft surgery within the last 3 months from the Visit 1
   3. Patients with a diagnosis of malignancy, including leukemia and lymphoma, within 5 years prior to Visit 1 (Patients who are assessed as having a complete response after treatment and who have not a relapsed for 5 years after the Visit 1 or those whose malignancy is well controlled basal cell carcinoma or squamous cell carcinoma of the skin can be enrolled)
   4. Patients with the following surgical or medical conditions that may affect absorption, distribution, metabolism and excretion of the medication
      1. Patients with hereditary conditions such as galactose intolerance, Lapp lactase deficiency, or glucose-galactose malabsorption
      2. Patients with a history of total gastrectomy, total colectomy, enterocolectomy, gastroenteroanastomosis, or Roux-en-Y gastric bypass
      3. Patients with a history of following gastrointestinal conditions (active inflammatory bowel disease, cholestasis, or atresia of bile ducts) within 12 months of Visit 1
      4. Patients with peptic ulcer, GI bleeding requiring treatment at Visit 1
   5. Patients with chronic medical conditions requiring continuous use of systemic steroids or immunosuppressive agents
   6. Patients with psychological problems not controlled by medication
   7. Patients with Cushing syndrome, AIDS, or secondary dyslipidemia
   8. Patients who are resistant or hypersensitive to HMG-CoA reductase inhibitor and Ezetimibe
   9. Patients with a history of drug or alcohol abuse within the year prior to the Visit 1
   10. Patients requiring concomitant contraindicated medications such as glecaprevir and pibrentasvir
   11. Patients who are pregnant or breastfeeding, or who are planning to become pregnant during the study, or who have not agreed to use an adequate method of contraception

**Supplementary Table 2. Risk category and LDL cholesterol goals**

| **Risk category^*^** | **LDL cholesterol goals (mg/dL)** |
| --- | --- |
| Low-risk group (at least one of the risk factor^**^) | <160 |
| Moderate-risk group (two or more risk factors) | <130 |
| High-risk group  - Carotid artery disease  - Abdominal aortic aneurysm  - Diabetes | <100 |
| Very high-risk group  - Coronary artery disease  - Atherosclerotic ischemic stroke and transient cerebral ischemic attack  - Peripheral artery disease | <70 |

^*^ Based on the Korean Guidelines for the Management of Dyslipidemia 4th edition

^**^The major risk of cardiovascular diseases except LDL-cholesterol

- Age: male ≥45 years, female ≥55 years (at Visit 1)
- Family history of premature atherosclerotic cardiovascular disease (male <55 years, female <65 years) (at Visit 1)
- Hypertension: SBP ≥140 mmHg or DBP ≥90 mmHg or taking antihypertensive drugs (at Visit 3)
- Smoking (at Visit 1)
- Low HDL-C: HDL-C <40 mg/dL (at Visit 2)
- High HDL-C (≥60 mg/dL) is considered as a protective factor, and one factor is excluded from the number of risk factors (at Visit 2)

**Supplementary Table 3. Changes in lipid profile at 4-week and 8-week follow-up**

|  | **Atorvastatin 5 mg/Ezetimibe 10 mg (n=61)** | | | | | **Atorvastatin 5 mg**  **(n=60)** | | | | | **Ezetimibe 10 mg**  **(n=63)** | | | | | **Atorvastatin 10 mg**  **(n=62)** | | | | |
| --- | --- | --- | --- | --- | --- | --- | --- | --- | --- | --- | --- | --- | --- | --- | --- | --- | --- | --- | --- | --- |
|  | Baseline | W4 | % change | W8 | % change | Baseline | W4 | % change | W8 | % change | Baseline | W4 | % change | W8 | % change | Baseline | W4 | % change | W8 | % change |
| **LDL-C, mg/dL** | 150.9  ±32.9 | 78.1  ±17.0 | -46.7  ±10.5* | 77.2  ±24.3 | -47.6  ±15.5* | 152.8  ±30.8 | 101.7  ±24.4 | -32.1  ±14.7* | 99.6  ±24.2 | -33.4  ±14.7* | 144.8  ±32.7 | 117.4  ±24.3 | -17.7  ±12.9* | 114.2  ±25.4 | -19.4  ±16.4* | 152.9  ±27.6 | 90.9  ±21.1 | -40.3  ±10.9* | 91.9  ±24.6 | -40.1  ±12.3* |
| **Total cholesterol, mg/dL** | 229.9  ±37.2 | 151.7  ±25.7 | -33.0  ±9.3* | 147.9  ±30.4 | -34.9  ±11.7* | 226.8  ±35.1 | 173.8  ±31.9 | -22.8  ±11.0* | 168.6  ±29.0 | -25.0  ±10.5* | 222.4  ±36.4 | 189.4  ±28.6 | -14.4  ±7.8* | 185.6  ±31.7 | -15.5  ±11.9* | 224.4  ±32.4 | 159.1  ±26.1 | -29.0  ±8.3* | 161.3  ±30.2 | -28.3  ±10.0* |
| **Triglycerides, mg/dL** | 190.1  ±93.0 | 135.1  ±60.9 | -23.7  ±25.5* | 130.8  ±62.2 | -25.7  ±24.0* | 168.2  ±76.7 | 152.6  ±114.2 | -7.3  ±42.6* | 132.5  ±56.8 | -17.2  ±25.7* | 188.9  ±102.3 | 163.1  ±94.7 | -5.1  ±39.5 | 167.2  ±168.7 | -6.7  ±52.8* | 157.6  ±69.6 | 118.3  ±51.1 | -20.7  ±31.4* | 129.4  ±56.1 | -12.2  ±38.8* |
| **HDL-C, mg/dL** | 52.3  ±16.6 | 55.3  ±17.5 | 6.4  ±15.4* | 54.7  ±16.2 | 6.0  ±16.0* | 49.9  ±12.3 | 51.5  ±12.9 | 4.2  ±14.0* | 52.0  ±11.7 | 5.9  ±16.3* | 50.2  ±11.6 | 51.3  ±11.3 | 3.1  ±12.1 | 50.8  ±11.0 | 3.0  ±14.3 | 51.0  ±10.1 | 53.8  ±11.0 | 5.9  ±15.4* | 53.8  ±10.4 | 6.4  ±16.0* |
| **Non-HDL-C, mg/dL** | 177.6  ±34.6 | 96.5  ±18.6 | -44.3  ±9.9* | 93.2  ±25.3 | -46.7  ±13.0* | 176.9  ±33.9 | 122.3  ±31.1 | -30.1  ±14.2* | 116.6  ±26.8 | -33.2  ±12.3* | 172.2  ±35.5 | 138.2  ±29.3 | -19.4  ±9.4* | 134.8  ±31.8 | -20.5  ±15.2* | 173.3  ±28.8 | 105.3  ±23.7 | -39.3  ±10.2* | 107.5  ±27.5 | -38.4  ±11.6* |
| **Apo AI, mg/dL** | 145.1  ±29.8 | 148.1  ±31.8 | 2.6  ±12.8 | 147.3  ±28.0 | 2.5  ±12.8 | 136.8  ±22.6 | 140.9  ±21.6 | 3.8  ±11.2* | 140.0  ±21.8 | 3.3  ±12.5* | 139.9  ±22.3 | 141.7  ±22.2 | 1.5  ±8.2 | 141.5  ±22.5 | 2.2  ±8.6* | 138.5  ±20.8 | 142.3  ±21.2 | 2.6  ±10.0 | 142.6  ±21.1 | 3.3  ±11.4* |
| **Apo B, mg/dL** | 122.7  ±23.0 | 74.3  ±12.1 | -38.0  ±9.5* | 73.5  ±16.8 | -39.2  ±12.3* | 122.3  ±22.9 | 87.6  ±18.5 | -27.5  ±12.3* | 85.5  ±17.8 | -29.4  ±11.2* | 117.7  ±23.3 | 100.0  ±19.7 | -14.7  ±9.0* | 97.3  ±19.5 | -16.3  ±13.0* | 120.4  ±18.9 | 79.0  ±14.3 | -34.6  ±8.9* | 80.2  ±16.4 | -33.6  ±9.4* |
| **Lipid parameters ratio** |  |  |  |  |  |  |  |  |  |  |  |  |  |  |  |  |  |  |  |  |
| **LDL-C/HDL-C** | 3.1  ±0.9 | 1.5  ±0.5 | -49.2  ±11.3* | 1.5  ±0.5 | -50.3  ±13.6* | 3.2  ±0.9 | 2.1  ±0.6 | -34.0  ±15.7* | 2.0  ±0.6 | -36.2  ±14.6* | 3.0  ±0.8 | 2.4  ±0.7 | -19.8  ±10.5* | 2.3  ±0.6 | -21.1  ±15.6* | 3.1  ±0.7 | 1.8  ±0.5 | -42.9  ±11.3* | 1.8  ±0.5 | -43.2  ±10.9* |
| **Total cholesterol/HDL-C** | 4.7  ±1.5 | 2.9  ±0.7 | -36.1  ±11.2* | 2.8  ±0.7 | -38.0  ±10.8* | 4.8  ±1.2 | 3.5  ±1.0 | -24.5  ±16.2* | 3.4  ±0.7 | -28.0  ±12.1* | 4.6  ±1.1 | 3.8  ±0.9 | -16.0  ±10.3* | 3.8  ±1.1 | -16.4  ±17.5* | 4.5  ±0.9 | 3.0  ±0.6 | -32.0  ±10.2* | 3.1  ±0.6 | -31.8  ±10.2* |
| **Non-HDL-C/HDL-C** | 3.7  ±1.2 | 1.9  ±0.7 | -46.6  ±13.0* | 1.8  ±0.7 | -49.0  ±12.8* | 3.8  ±1.2 | 2.5  ±1.0 | -31.1  ±20.2* | 2.4  ±0.7 | -35.6  ±14.7* | 3.6  ±1.1 | 2.8  ±0.9 | -20.7  ±12.9* | 2.8  ±1.1 | -21.0  ±22.2* | 3.5  ±0.9 | 2.0  ±0.6 | -41.5  ±12.8* | 2.1  ±0.6 | -41.2  ±12.7* |
| **Apo B/Apo AI** | 0.9  ±0.2 | 0.5  ±0.1 | -38.8  ±11.6* | 0.5  ±0.2 | -40.0  ±13.6* | 0.9  ±0.2 | 0.6  ±0.2 | -29.4  ±14.3* | 0.6  ±0.2 | -30.7  ±13.2* | 0.9  ±0.2 | 0.7  ±0.2 | -15.6  ±9.6* | 0.7  ±0.2 | -17.8  ±13.7* | 0.9  ±0.2 | 0.6  ±0.1 | -35.5  ±9.7* | 0.6  ±0.1 | -35.5  ±8.0* |
| **hs-CRP, mg/dL** | 1.2  ±1.1 | 1.7  ±4.0 | 51.8  ±190.0 | 2.8  ±10.2 | 63.6  ±290.9 | 1.8  ±4.4 | 1.5  ±2.7 | 162.2  ±692.2 | 1.2  ±2.0 | 11.1  ±95.9 | 1.3  ±1.9 | 1.5  ±1.9 | 65.3  ±217.8 | 1.7  ±2.5 | 57.8  ±238.3 | 1.1  ±1.2 | 1.1  ±1.4 | 41.7  ±232.7 | 1.4  ±2.9 | 78.5  ±358.2 |
| **Fibrinogen, mg/dL** | 290.7  ±69.3 | 294.1  ±67.7 | 4.7  ±27.6 | 295.1  ±74.8 | 3.6  ±22.3 | 311.7  ±79.9 | 281.1  ±55.0 | -6.3  ±20.3* | 288.2  ±73.8 | -4.4  ±21.8* | 286.8  ±65.8 | 279.2  ±70.0 | -0.6  ±25.9 | 287.0  ±79.8 | 0.2  ±21.1 | 301.7  ±66.1 | 282.2  ±78.4 | -4.3  ±27.2 | 281.6  ±68.8 | -4.9  ±22.6 |

Data presented as mean ± standard deviation or as n (%). *In group P-value <0.05.

Abbreviations: Apo AI, apolipoprotein A-I; Apo B, apolipoprotein B; HDL-C, high density lipoprotein cholesterol; LDL-C, low density lipoprotein cholesterol; non-HDL-C, non–high-density lipoprotein cholesterol.

**Supplementary Table 4. Treatment-related side effects by events**

| **Characteristics** | **Atorvastatin 5 mg/Ezetimibe 10 mg**  **(n=62)** | **Atorvastatin 5 mg**  **(n=61)** | **Ezetimibe 10 mg**  **(n=63)** | **Atorvastatin 10 mg**  **(n=64)** |
| --- | --- | --- | --- | --- |
| **Treatment emergent adverse event (events)** | 14 | 12 | 9 | 16 |
| Mild | 13 | 11 | 7 | 16 |
| Moderate | 1 | 0 | 1 | 0 |
| Severe | 0 | 1 | 1 | 0 |
| **Adverse drug reaction (events)** | 2 | 1 | 1 | 4 |
| Gastrointestinal disorders | 1 | 0 | 1 | 1 |
| Myalgia | 0 | 1 | 0 | 1 |
| Dizziness | 0 | 0 | 0 | 2 |
| Creatine phosphokinase increased | 1 | 0 | 0 | 0 |
| **Serious adverse event (events)** | 0 | 1 | 1 | 0 |
| Ulna fracture | 0 | 1 | 0 | 0 |
| Spinal pain | 0 | 0 | 1 | 0 |
| **Adverse event leading to withdrawal (events)** | 1 | 0 | 0 | 1 |
| Creatine phosphokinase increased | 1 | 0 | 0 | 0 |
| Dizziness | 0 | 0 | 0 | 1 |

Multiple events occurring in the same patient were counted separately.


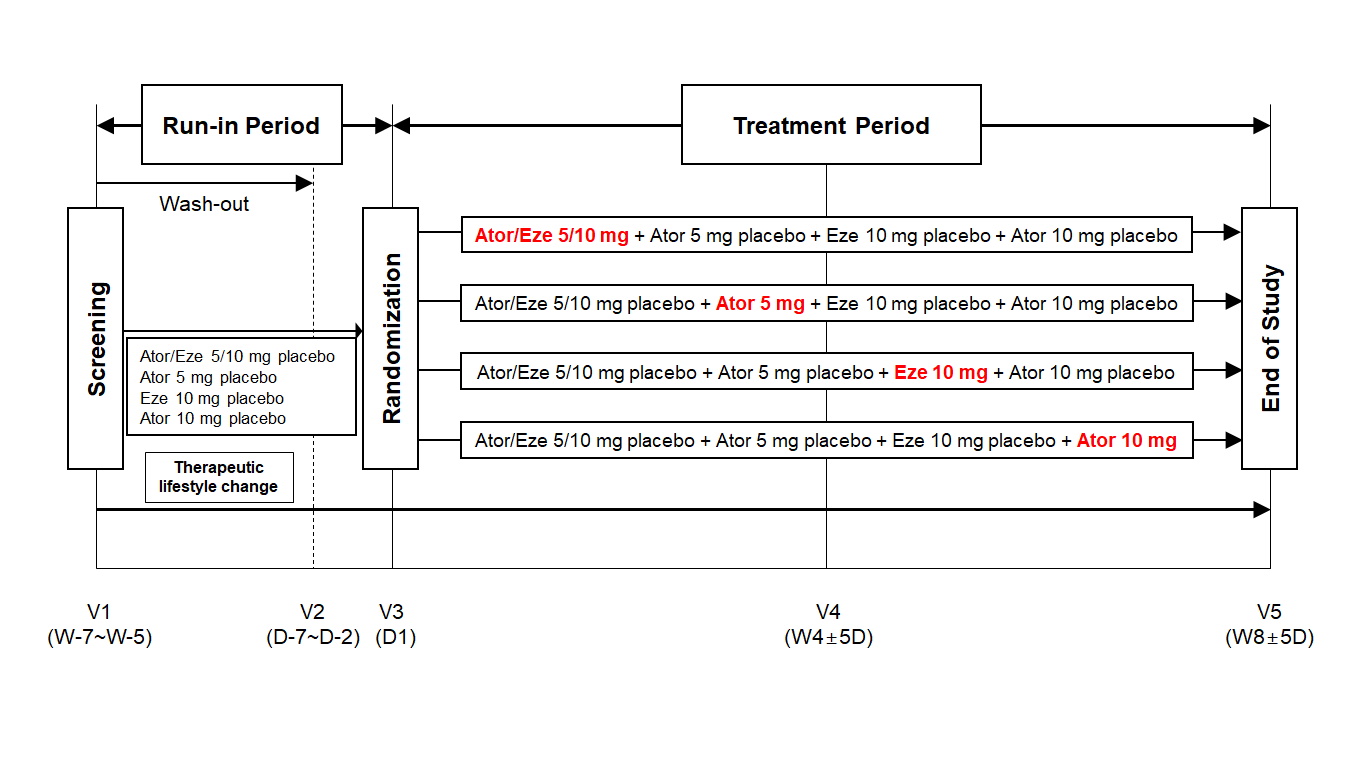


**Figure S1. Study protocol**

Ator, atorvastatin; Eze, ezetimibe.


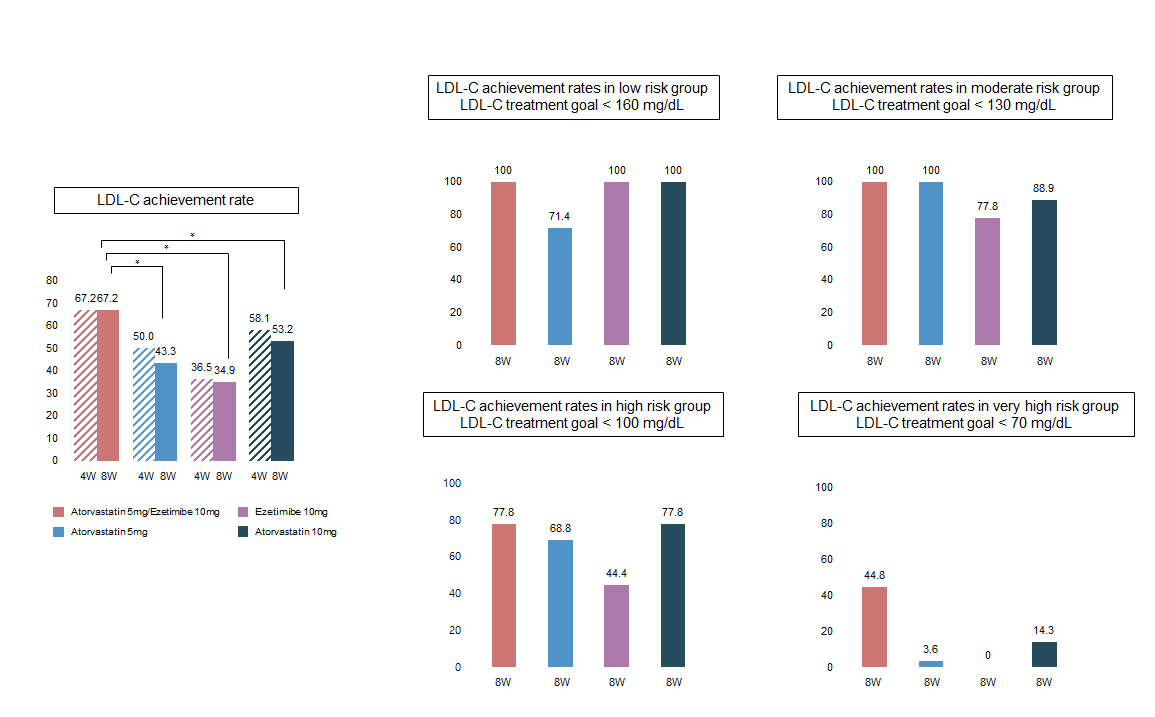


**Figure S2. Percentage of patients achieving target low-density lipoprotein cholesterol by cardiovascular disease risk category**

^*^Between group P-value <0.0001

LDL-C, low density lipoprotein cholesterol.
